# Editorial: Advances in Cancer Diagnosis and Treatment

**DOI:** 10.3389/bjbs.2024.13618

**Published:** 2024-09-06

**Authors:** Mark O. R. Hajjawi, Qiuyu Wang, Nadege Presneau, Michael R. Ladomery

**Affiliations:** ^1^ Blood Sciences Department, York and Scarborough Teaching Hospitals NHS Foundation Trust, York, United Kingdom; ^2^ Department of Life Sciences, Manchester Metropolitan University, Manchester, United Kingdom; ^3^ School of Life Sciences, University of Westminster, London, United Kingdom; ^4^ Faculty of Health and Applied Sciences, University of the West of England, Bristol, United Kingdom

**Keywords:** cancer biomarkers, cancer diagnosis, cancer treatment, novel diagnostics, novel biomarkers, oncology, cancer test

This Special Issue of the *British Journal of Biomedical Science* brings together a collection of articles which all contribute to the advancement of knowledge into the diagnosis and treatment of cancer. Each article in this diverse research field offers an insight into current clinical practice or provides evidence for a potential future novel diagnostic test or treatment. Each manuscript presented here has the potential to contribute directly towards improving patient outcomes.

Cancer is a burden on society across the world. Cole et al. presented work focusing on colorectal cancer within the United Kingdom, while Liau et al. conducted work focusing on colorectal cancer in Malaysia. Both groups of authors highlighted that colorectal cancer in their studied populations has a high prevalence and mortality rate.

Cole et al. reviewed the clinical utility of one of the current tests used in the screening and diagnosis of colorectal cancer. The faecal immunochemical test (FIT) detects haemoglobin in a person’s faeces and is used to determine if occult bleeding is present in the gastrointestinal tract and whether further invasive follow-on testing is required. Studying a large population in the north of England, Cole et al. presented evidence showing that 8% of FIT tests in people later confirmed to have colorectal cancer were incorrectly classified as negative. The authors point out that this only accounts for 0.06% of all FIT performed in this population, and that the test still has a very high diagnostic sensitivity, but highlight the need for improving diagnostic tests.

The review by Liau et al. on colon cancer-associated transcript-1 (CCAT-1) goes some way towards providing pleasing evidence for a new diagnostic test for colorectal cancer. This long non-coding RNA (lncRNA) was originally found to be over-expressed in colorectal cancer, but has now been shown to be over-expressed in many types of cancers. Liau et al. are keen to stress that there is a considerable amount still not known about CCAT-1, including its mechanism of action and factors that cause its dysregulation, but highlight that its downregulation may be correlated with drug sensitivity and better treatment outcomes.

Continuing the theme of manipulating a patient’s RNA to improve chemotherapy drug sensitivity, Wodi et al. demonstrated the potential of a novel therapeutic in the Kasumi-1 cell line model of acute myeloblastic leukaemia. Wodi et al. used a 3-(trifluoromethyl)anilide scaffold named SPHINX to inhibit the activity of the splice factor protein kinase in a cell culture model of leukaemia, thereby manipulating alternative splicing. This splice factor protein kinase regulates the activity of SRSF1, an important SR protein splice factor regulator of alternative splicing of many critical cancer-associated genes. Alternative splicing, in which one multi-exon gene can produce many different mRNA transcripts, via skipping or retaining introns and exons, is a widespread process that accounts for some of the complexity of the proteome. Dysregulated alternative splicing is a feature of many cancers, and the authors present compelling evidence that splice factor kinase inhibition could bring therapeutic benefits.

Remaining on the theme of cancers of the blood and bone marrow, Al-Zubaidi and Hughes investigated the role of a new biomarker using immunophenotyping flow cytometry to help differentiate between different B-cell lymphoproliferative disorders. The authors begin with a succinct overview of the World Health Organisation classification of B-cell lymphoproliferative disorders and summarised the difficulties in distinguishing between these heterogeneous leukaemias and lymphomas. Highlighting how an atypical presentation of chronic lymphocytic leukaemia can cause diagnostic uncertainty, the authors presented further evidence for the role of CD200 in diagnostic algorithms. The authors outline the case to give further support for including CD200 in routine immunophenotyping testing panels.

Focusing on white blood cells of the myeloid lineage, Chohan et al. presented a meta-analysis of oral squamous cell carcinoma (OSCC) and biomarkers that may be useful in determining prognosis. Focusing on tumour associated macrophages and the role they play in OSCC, the authors investigated the role of CD68 and CD163 expression by these cell types along with PD-L1. The authors present evidence suggesting that CD163 positive tumour associated macrophages were connected with a poor prognosis in OSCC, but CD68 positive macrophages had no correlation with prognosis. The authors also found that raised PD-L1 may have a positive impact on prognosis, but warn that whether this expression is located in the tumour or stromal cells may be a significant factor. However, the authors conclude that the available evidence is currently too weak to support claims on the utility of PD-L1. The authors completed their analysis by contrasting their findings in OSCC to the results seen in studies of other cancers.

Murugan and Alzahrani analysed almost 15,000 solid malignancy samples across 37 cancer types stored in the cancer genome atlas, looking for mutations in the genes for isocitrate dehydrogenases 1 and 2. The authors found that approximately 3% of cancers overall contained mutations in isocitrate dehydrogenases 1 (*IDH1*), but this rose to 34% in gliomas. Linking these mutations to prognosis, the authors presented evidence to show that patients with a mutation in *IDH1* had an improved overall survival rate and better progression free survival. The authors conclude their paper by linking their work on mutant *IDH1* and 2 with the effectiveness of the inhibitors ivosidenib and enasidenib, used in the treatment of acute myeloid leukaemia. They hypothesise that there may also be a key therapeutic potential in targeting *IDH1* and 2 in gliomas and other malignancies.

These six papers, although different in subject areas, aims and methodologies, each contribute to the ongoing development of new diagnostics and treatments for cancer.

